# Integrated models of care for diabetes and hypertension in low- and middle-income countries (LMICs) : Protocol for a systematic review

**DOI:** 10.1186/s13643-018-0865-8

**Published:** 2018-11-20

**Authors:** Jeannine Uwimana Nicol, Anke Rohwer, Taryn Young, Charlotte M Bavuma, Joerg J Meerphol

**Affiliations:** 1 Centre for Evidence-Based Health Care, Division of Epidemiology and Biostatistics, Department of Global Health, Faculty of Medicine and Health Sciences, Stellenbosch University, Francie van Zijl drive, Parow, Cape Town, 7500 South Africa; 2 School of Public Health, College of Medicine and Health Science, University of Rwanda, Kicukiro, Kigali, Rwanda; 3 College of Medicine and Health Science, University of Rwanda, Kicukiro, Kigali, Rwanda; 4 Institute for Evidence in Medicine (for Cochrane Germany Foundation), Medical Center–University of Freiburg, Breisacher Strasse 153, 79110 Freiburg, Germany

**Keywords:** Non-communicable diseases, Diabetes, Hypertension, Multi-morbidity, Integrated care, Low- and middle-income countries

## Abstract

**Background:**

In low- and middle-income countries (LMICs), the burden of non-communicable diseases (NCDs) is growing against an existing burden of other diseases such as HIV/AIDS. Integrated models of care can help address the rising burden of multi-morbidity. Although integration of care can occur at various levels and has been defined in numerous ways, our aim is to assess the effects of integration of service delivery at primary healthcare level in LMICs.

**Methods:**

We will consider randomised controlled trials (RCTs), cluster RCTs, non-randomised trials, controlled before-after studies and interrupted time series that examine integrated models of care among people with multi-morbidities, of which diabetes or hypertension is one, living in LMICs. We will compare fully integrated models of care to stand-alone care, partially integrated models of care to stand-alone care and fully integrated models to partially integrated models of care. Primary outcomes include all-cause mortality, disease-specific morbidity, HbA1c, systolic blood pressure and cholesterol levels. Secondary outcomes include access to care, retention in care, adherence, continuity of care, quality of care and cost of care. We will conduct a comprehensive search in the following databases: MEDLINE, EMBASE, the Cochrane Central Register of Control Trials, LILACS, Africa-Wide Information, CINAHL and Web of Science. In addition, we will search trial registries, relevant conference abstracts and check references lists of included studies. Selection of studies, data extraction and assessment of risk of bias will be performed independently by two review authors. We will resolve discrepancies through discussion with a third author. We will contact study authors in case of missing data. If included studies are sufficiently homogenous, we will pool results in a meta-analysis. Clinical heterogeneity related to the population, intervention, outcomes and context will be documented in table format and explored through subgroup analysis. We will assess *χ*^*2*^and *I*^2^ tests for statistical heterogeneity. We will use GRADE to make judgements about the certainty of evidence and present findings in a summary of findings table.

**Discussion:**

In light of limited evidence on the provision of comprehensive care for diabetes and hypertension, and its comorbidity in LMCIs, we believe that the findings of this systematic review will provide a synthesis of evidence on effective models of integrated care for diabetes and hypertension and their comorbidities at primary healthcare level. This will enable policy-makers to device policies and programs that are evidence informed.

**Systematic review registration:**

PROSPERO CRD42018099314.

**Electronic supplementary material:**

The online version of this article (10.1186/s13643-018-0865-8) contains supplementary material, which is available to authorized users.

## Background

The burden of non-communicable disease (NCDs) in low- and middle-income countries (LMICs) exceeds that of high-income countries [1]. In 2015, a total of 40 million people in LMICs died of NCDs (80% of deaths) of which 15 million deaths were premature and occurred between the ages of 30–70 years, the prime productive age of employment in most LMICs [1, 2]. This implies that NCDs are a threat to the health of adults in LMICs, to workplace productivity and to the health of economies [3].

NCDs are an umbrella term referring to cardiovascular diseases (heart attacks and stroke), cancers, chronic respiratory diseases (such as chronic obstructive pulmonary disease and asthma) and diabetes. Most NCD deaths are caused by cardiovascular diseases (17.7 million deaths per year) followed by cancers (8.8 million deaths per year), respiratory diseases (3.9 million deaths per year) and diabetes (1.6 million deaths per year) [1]. NCDs account for the majority of illness in all regions of the world other than sub-Saharan Africa.

Although exposure to risk factors is the main cause of premature NCD deaths, an inadequate response to the healthcare needs of affected patients contributes a great deal [4]. Diabetes and hypertension are the major cardiovascular risks leading to target organs’ damage such as the brain, heart and kidney. Currently, the number of people with diabetes is estimated to be 425 million and it is expected to increase up to 629 million in 2045, and its burden is reported to be serious in productive age [5]. Similarly, the prevalence of hypertension is increasing worldwide particularly in LMICs. According to the International Society of hypertension (ISH), around 40% of people over age of 25 years have hypertension worldwide and two third of them live in LMICs [6]. In addition to standalone burden, hypertension and diabetes co-morbidity have been reported to have a synergistic impact on bad cardiovascular disease outcomes [7].

### Multi-morbidities of NCDs

Multi-morbidity is defined as the presence of two or more chronic medical conditions in an individual [8]. Multi-morbidity of NCDs poses a global healthcare challenge. In most LMICs, co-morbidity of NCDs and communicable diseases (CDs) is increasing [9, 10]. The interaction between NCDs and CDs, particularly with human immunodeficiency virus (HIV) and its treatment with cardio-metabolic disorders, and between smoking, diabetes and tuberculosis (TB) has been highlighted in the literature [10, 11].

Due to improved treatment programs for HIV, people living with HIV (PLWH) are living longer with an increasing risk of developing NCDs due to direct nature of HIV infection, shared risk factors and treatment side effects of HIV [10–12].

Antiretroviral treatment (ART) of HIV infection increases the risk of hyperlipidaemia and diabetes. In addition, PLWH have a high risk of contracting NCDs through the risk associated with increasing age [9, 13].

Moreover in LMICs, HIV remains a major threat among vulnerable groups and particularly in some parts of Africa where HIV and AIDS are the greatest cause of life-years lost [14, 15].

Beside the epidemiological link between NCDs and CDs, studies in the literature highlight interaction between NCDs and psychological disorders such as depression [16, 17].

A study conducted in Nigeria shows that the prevalence of depression was 28% in diabetic patients and 27% in patients with hypertension [18]. In light of multi-morbidity of NCDs in particular diabetes and cardiovascular diseases, it is imperative for LMICs to have a strong and dynamic health system that can respond effectively to the growing burden of multi-morbidity.

### Management of NCDs

In 2010, the WHO published a Package of Essential NCDs (PEN) interventions for primary healthcare (WHO PEN) to assist low-income countries for early detection and management of NCDs at primary healthcare (PHC) level [19]. WHO PEN, provides a package of set cost-effective interventions to be delivered at low cost and acceptable quality of care, but it does not indicate how these interventions can be delivered in an integrated manner with other chronic diseases such as HIV [19]. Hence, NCDs such as diabetes and hypertension are still managed separately from other chronic diseases such as TB and HIV [20].

In order to curtail the burden of NCDs and its co-morbidities, LMICs should consider accelerating the provision of comprehensive care and integrated strategies to improve health service delivery, efficiency and equity [13].

A paradigm shift from vertical programs towards integrated approach is required to address shared NCDs risks and socio-economic determinant factors [9, 21]. Haldane and colleagues argue that integrated models of care provide people with holistic options centred on health needs of people and communities and thereby enhance community self-reliance [21].

### Need for integrated approach for management of NCDs

Given the multiple comorbidities of hypertension and diabetes, they require comprehensive care that is health-centred rather than disease-tailored, particularly in LMICs where both the burden of NCDs and CDs is rising [13]. In addition, LMICs need health systems that can respond more effectively and equitably to the healthcare needs of people with NCDs in order to reduce morbidity, disability and death from NCDs, and contribute to better overall health outcomes [4].

The term *integration* is mostly defined in the literature as “managerial or operational changes to health systems to bring together inputs, delivery, management and organization of particular service functions as means of improving coverage, access, quality, acceptability and cost-effectiveness” [22]. This may include service integrations that combine “different packages of services such as integration of service delivery points, integration at different levels of service delivery, process modifications, introduction of technologies aimed at aiding integration, and integration of management decisions” [23, 24].

Lately, most policy-makers and researchers advocate for integrated approach or programs because integrated programs increase system effectiveness and cost-effectiveness, particularly in low-resource settings [13, 25, 26].

Curry and Ham (2012) as well as Valentijn and colleagues (2013) propose typologies of integrated care. This typology differentiates  integration at micro-level as patient focused, such as case management, whereas meso-level integration focuses on groups of patients or population, and macro-level integration focuses on health systems [23, 24]. Within these levels, the typology distinguishes integration types.

We classify clinical integration as care integrated into a single process through shared guidelines and protocols across professions, and service integration as different clinical services integrated within an organisation and provided through single healthcare worker or multidisciplinary teams.

**Fig. 1:**
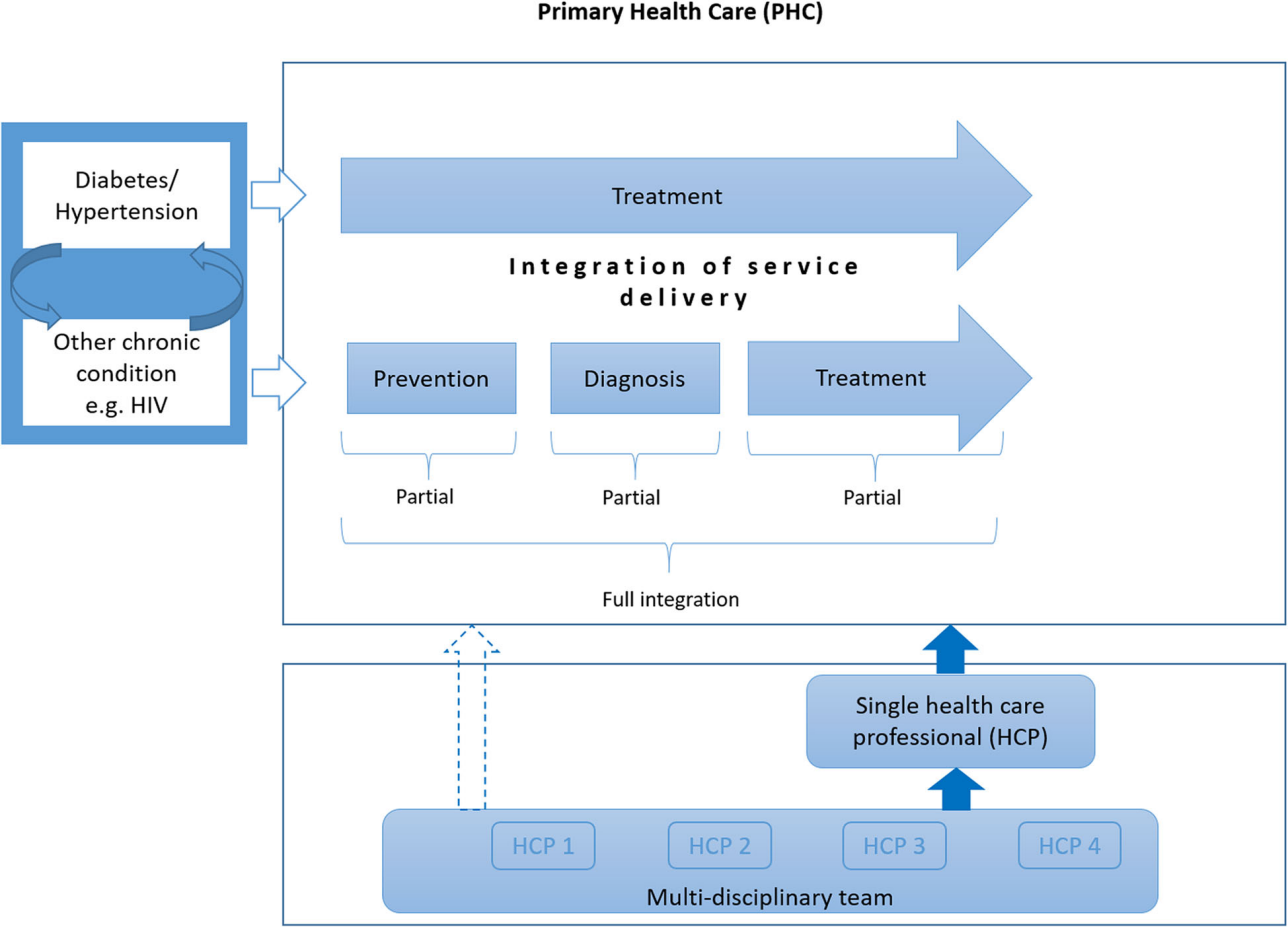
Logic model of models of integrated care for diabetes and hypertension

In recent years, guidelines, as well as research from LMICs (and sub-Saharan Africa in particular), have focused on integration of HIV/AIDS and TB services at service delivery level of the health system [15, 27, 28]. Drawing on these and other models of care, we have conceptualised integrated care as partial integration and full integration of service delivery as illustrated in a logic model of integrated care for diabetes and hypertension (Fig. 1). In this model, we assume that a patient has already entered the health system (i.e. through a PHC clinic and or community level) either for treatment of diabetes or hypertension, or for treatment of any other chronic disease such as HIV.

In a fully integrated model of service delivery, a patient with more than one chronic disease should receive the full package of care for all diseases at a single point of care. This “one-stop-shop” model refers to horizontal integration of primary healthcare and community health services and can involve one or more healthcare professionals. In a partially integrated model of service delivery, patients receiving treatment for one disease receive additional care related to prevention, diagnosis or treatment of another disease, but do not receive the full package of care (Fig. 1).

### Rationale for systematic review

We conducted a scoping review (date of last search 8 November 2016) to assess existing evidence on the effectiveness of integrated models of care in people with diabetes or hypertension and any other comorbid disease [29]. We identified five systematic reviews, of which two examined collaborative care for diabetes and depression, two looked at collaborative care for various diseases including diabetes and depression, hypertension and diabetes, and hypertension and depression; and one focused on task-shifting and management of cardiovascular diseases and diabetes [29]. However, none of the included reviews examined collaborative care for diabetes or hypertension and communicable diseases, and only one review that assessed task-shifting included studies from LMICs.

Subsequently, another review by Haldane et al. [21] examined existing programmes that integrated healthcare delivery for diabetes, hypertension or CVDs with HIV/AIDS (date of last search October 2015). They included 17 studies of which most described existing programmes and eleven of these described programmes were in sub-Saharan Africa. Although this is useful for planning and development, there is a lack of data on the effectiveness of these programmes.

In addition, little was found on factors that facilitate or hinder integration of hypertension and diabetes with co-morbidities in LMICs. Another descriptive study from Cambodia on the management of HIV/AIDS, diabetes and hypertension showed that integration of these services was highly acceptable and led to good health outcomes [30].

A Cochrane review by Dudley and Garner assessed the effectiveness of strategies to integrate primary healthcare services in LMICs [31]. They included studies that integrated family planning into existing services; nutrition and infectious disease interventions; and sexually transmitted infections, HIV/AIDS and TB treatment. None of the included studies reported on services linked to NCDs.

There is thus little up-to-date evidence from LMICs on the effectiveness of integrated healthcare delivery for people with NCDs, and diabetes and hypertension in particular. With a rising burden of diabetes and hypertension, in combination with the existing high burden of infectious diseases and resource constrained health systems in LMICs, there is a need to optimise healthcare delivery to benefit both the patients and the health systems.

The objective of this systematic review is to assess the effects of integrated models of care at PHC level for people living in LMICs, with multi-morbidity, of which diabetes or hypertension is one, compared to no integrated care on health and process outcomes.

## Methods

This protocol is reported according to the PRISMA-P checklist [32] (Additional file 1).

### Criteria for considering studies for the review

- *Types of participants*

We will include studies examining people with multi-morbidity, of which diabetes and/or hypertension is one, living in LMICs, irrespective of age. We define multi-morbidity as the presence of two or more chronic medical conditions in an individual [8]. Studies addressing both adults and children will be considered. LMICs will be defined according to the classification of the World Bank [33].

- *Types of interventions*

This review will only consider studies that describe integration of service delivery at PHC and community level. We will consider models of partial integration and full integration of service delivery (Fig. 1). Partial integration of service delivery will be defined as models where patients treated for diabetes, hypertension or any other chronic disease receive part of the package of care (prevention, diagnosis, treatment) for another disease. Full integration of service delivery will be defined as models where patients (primarily treated for diabetes, hypertension or any other disease) receive the full package of care (prevention, diagnosis and treatment) for diabetes/hypertension and any other chronic disease at the same point of care by one or more healthcare professionals.

- *Types of comparisons*

The main comparison will be stand-alone models of care, defined as models of care that are limited to one disease. We will include the following comparisons:

- ◦ Fully integrated models of care vs. stand-alone care
- ◦ Partially integrated models of care vs. stand-alone care
- ◦ Fully integrated models of care vs. partially integrated models of care

- *Types of outcomes*

We will include studies that report on either primary health outcomes or secondary outcomes. However, the absence of reporting the pre-specified health and process outcomes will not be a deciding factor for inclusion of studies. Types of outcomes to consider are the following:

### Primary outcomes (health outcomes)

- ▪ All-cause mortality
- ▪ Disease specific morbidity as reported in included studies (e.g. disease control metrics, quality of life)
- ▪ Glycated haemoglobin (HbA1c)
- ▪ Systolic blood pressure (BP)
- ▪ Cholesterol levels

### Secondary outcomes (process outcomes)

- ▪ Access to care as reported in the included studies
- ▪ Retention in care and adherence as reported in the included studies
- ▪ Continuity of care as reported in the included studies
- ▪ Quality of care as reported in the included studies
- ▪ Cost of care as reported in the included studies

- *Types of studies*

We will consider randomised controlled trials (RCTs), including cluster RCTs, controlled (non-randomised) clinical trials (CCTs) or cluster trials, interrupted time series (ITS) studies with at least three data points before and after the intervention, and controlled before-and-after (CBA) studies for inclusion. Cluster randomised, cluster non-randomised or CBA studies will be included only if there are at least two intervention sites and two control sites. Cross-sectional studies, case series and case reports will be excluded.

### Search methods for identifying studies

We will search the following electronic databases: MEDLINE (PubMed), EMBASE (Ovid), the Cochrane Central Register of Control Trials (CENTRAL), LILACS, Africa-Wide Information (via EBSCO host), CINAHL, Web of Science (Core collection). For ongoing studies, we will search the following trial registries: WHO ICTRP and Clinicaltrials.gov. We will search conference abstracts from the International AIDS Society Online Resource Library, the HIV/AIDS Implementers’ Meetings and the NCDs Alliance meetings.

Search terms will include ‘diabetes’, ‘hypertension’, ‘comorbidities , ‘integrated healthcare delivery’, ‘low- and middle-income countries’ and their synonyms. The full search strategy for MEDLINE (PubMed) is provided in Additional file 2. We will adapt it for other electronic databases. We will report all search strategies in full in the final version of the review. In addition, we will screen reference lists of included studies and reference lists of relevant systematic reviews, and contact experts in the field and relevant organisations (e.g. NCD Alliance) for unpublished studies. All languages will be included.

### Study selection and data extraction

- *Selection of studies*

Two authors will independently screen titles and abstracts of studies identified by the search using Covidence software, and we will retrieve full-text of all potentially eligible studies. Two authors will independently screen full texts for eligibility. Discrepancies in the selection process will be resolved through discussion or by consulting a third author. Studies will be classified as included, excluded or awaiting assessment. We will provide reasons for excluding studies. Studies only available as abstracts will be included in the general results of the review, but not in the analysis.

- *Data extraction*

We will extract data based on the description of various models of integrated care as illustrated in Fig. 1 with consideration of possible scenarios of various packages of care provided in terms of partial integration. A pre-specified, standardised and piloted data extraction form will be used. Two authors will independently extract data and compare the results. Discrepancies will be resolved by discussion or by consulting a third reviewer. We will contact study authors in case of missing data. We will extract data on the participants, intervention, comparisons, outcomes, setting, context and funding sources.

Data items will be in line with recommendations from the template for intervention description and replication (TIDieR) [34] and the PRISMA-Complex Interventions (CI) extension checklist [35]. We will look at the following items:

- ▪ Provide name or a phrase to describe the intervention
- ▪ Rationale, theory or goal of the element essential to the intervention
- ▪ Materials used
- ▪ Procedures or processes used in the intervention
- ▪ Who provided—each category of the intervention provider
- ▪ Describe modes of delivery of the intervention
- ▪ Types of location where the intervention took place
- ▪ When and how much—number of times the intervention was delivered
- ▪ Tailoring of the intervention
- ▪ How well the intervention was planned
- ▪ Replicability

For the ‘implementation components’, we will look at the following items:

- ▪ Clearly define the adoption, uptake or integration strategies

Integration strategies may include facilitators (distinct from intervention elements) such as attestations, financial incentives, periodic reports of findings, reminders, supplemental trainings or physical environmental changes.

Where there is insufficient detail reported in the study, we will contact the study investigators for clarity or more information on the study. Disagreements will be resolved first by discussion and then by consulting at third author for consensus.

- *Risk of bias assessment*

For randomised controlled trials, non-randomised trials and controlled before-after studies, we will use the tool proposed by the Effective Practice and Organisation of Care (EPOC) group [36]. We will assess the following nine domains as having ‘low risk’, ‘high risk’ or ‘unclear risk’ of bias: (1) random sequence generation, (2) allocation concealment, (3) baseline outcome measurements, (4) baseline characteristics, (5) incomplete outcome data, (6) knowledge of allocated intervention (blinding), (7) protection against contamination, (8) selective outcome reporting and (9) other risks of bias. For cluster RCTs, we will assess additional risk of bias linked to recruitment, baseline differences, loss of clusters, incorrect analysis and compatibility with RCTs randomised by individuals. For ITS studies, we will also use the tool proposed by EPOC to assess whether: (1) the intervention was independent of other changes, (2) the shape of the intervention effect was pre-specified, (3) the intervention was unlikely to affect data collections, (4) knowledge of the allocated intervention was adequately prevented during the study, (5) incomplete outcome data was likely to bias results, (6) outcomes were reported selectively and (7) there were any other risks of bias. For each domain, risk of bias will be assessed as low, high or unclear. Two authors will independently assess risk of bias of included studies. We will resolve discrepancies through discussion or consulting a third author.

### Data analysis

- *Measures of treatment effect*

We will extract relevant outcome data for each study and enter it into Review Manager 5. For dichotomous outcomes, we will calculate risk ratios (RR) and report pooled effects with 95% confidence intervals. For continuous outcomes, we will calculate the mean differences (MD) if outcomes were measured in the same way across studies or standardised mean differences (SMD) where outcomes were measured differently across studies. We will report pooled effects with 95% confidence intervals.

- *Unit of analysis issues*

Where cluster RCTs have appropriately adjusted for the effects of clustering in their analysis, we will use these adjusted effect estimates and standard errors in our meta-analysis using the generic inverse-variance method in Review Manager 5 [37]. Where the included cluster RCTs did not perform any adjustment for clustering, we will adjust the raw data ourselves using the intra-class correlation coefficient (ICC). If the study authors do not report an ICC value in the published article, either we will obtain this value from similar studies or we will estimate the ICC value. We will not present results from cluster RCTs that were not adjusted for clustering. If we estimate the ICC value, we will perform sensitivity analyses to investigate the robustness of our analyses. Where multi-arm studies (e.g. two intervention arms and one control arm) contribute multiple comparisons to a specific analysis, we will split the ‘shared group’ to avoid including data from the same participant more than once.

- *Dealing with missing data*

We will contact the study authors to request missing data if needed. If after contacting the authors, there are still missing data and we consider the data to be missing at random, we will include only the data available in the analysis. Otherwise, we will impute the missing data and account for the data imputed with uncertainty in line with the Cochrane Handbook of Systematic Reviews of Interventions [38]. We will then conduct a sensitivity analysis to analyse how sensitive the results are to the assumptions we made when imputing missing data and analyse all data as intention-to-treat.

- *Assessment of heterogeneity*

We will explore clinical heterogeneity by clearly documenting study characteristics related to the population, intervention, outcomes and context in table format. We will assess the statistical heterogeneity in each meta-analysis by inspecting forest plots and calculating *χ*^*2*^ test values and *I*^2^ statistics. We will consider significant heterogeneity present if the *P* value of the *χ*^*2*^ test is < 0.10. We will interpret the *I*^2^ statistic according to the thresholds recommended in the Cochrane Handbook of Systematic Reviews of Interventions [39]. Therefore, we will consider an *I*^2^ value above 30% to indicate important heterogeneity. In addition, we will explore the causes of statistical heterogeneity by conducting subgroup analyses.

- *Assessment of reporting biases*

We will examine reporting biases by means of funnel plots, if we are able to pool more than 10 studies per outcome in a meta-analysis.

- *Data synthesis*

We will organise the review findings according to the proposed models of integrated care as depicted in Fig. 1. We will pool data from individual studies if they are sufficiently homogeneous in terms of design, population, intervention and comparator. As we anticipate some degree of heterogeneity, we will perform random-effects meta-analysis. We will not pool data from RCTs and non-randomised studies in a single meta-analysis. If we judge included studies to be too heterogeneous to pool, we will make use of narrative synthesis and present data in table format.

- *Subgroup analysis*

We will carry out the following subgroup analyses on primary outcomes to explore heterogeneity: various co-morbidities (e.g. patients with diabetes and HIV vs patients with diabetes and depression), clinic vs. community level and age category (children, age 1–10 years vs. adolescents, age 10 to 19 years vs. adults, age > 19 years).

- *Sensitivity analysis*

We will carry out the following sensitivity analyses on primary outcomes:

- To examine the effect of excluding studies of high risk of bias
- To examine the effect of various ICCs in case of adjusting outcomes for clustering ourselves
- To examine the effect of imputed data

- *Summary of findings table and certainty of evidence*

We will assess the certainty of the evidence for primary outcomes (all-cause mortality, disease-specific morbidity, HbA1c, BP, cholesterol levels) using GRADE. We will create a ‘summary of findings’ table using GRADEpro software [40]. In the table, we will display the model of integrated care, primary outcomes (e.g. all-cause mortality, disease-specific morbidity) of the review, the comparative risks between intervention and control groups, the relative effects with 95% CI, the number of participants in the studies and the certainty of evidence.

The five domains that we will consider for our judgement to downgrade the certainty of evidence comprise study limitations, inconsistency, imprecision, indirectness and publication bias. We will consider upgrading the certainty of evidence if there is a large effect, a dose-response and cases where all plausible residual confounding would reduce a demonstrated effect or would suggest a spurious effect if no effect was observed. The quality of evidence for each outcome will be described as high, moderate, low or very low [41].

## Discussion

Given the rising burden of NCDs in light of communicable diseases in LMICs, policy-makers and healthcare professionals are seeking ways to provide comprehensive care to people with multi-morbidity. However, there is a lack of evidence on the effectiveness of integrated models of care in LMICs, hindering implementation of such models. Our systematic review aims to address this gap in evidence by providing an up-to-date and comprehensive synthesis of existing research. We will ensure methodological rigour by conducting an extensive search, systematically screening and selecting studies, extracting data and assessing the risk of bias of included studies. Furthermore, we will use GRADE to assess the quality of evidence. We have been engaging with policy-makers from the onset of this protocol, have involved them in the process of defining and clarifying the review question. We are also planning to continue this engagement throughout the review process. Hence, we believe that the findings of this systematic review will be relevant to policy-makers and inform policy and practice in LMICs.

## Additional files

Additional file 1: PRISMA-P checklist. (PDF 159 kb)

Additional file 2: Search strategy for MEDLINE (PubMed). (PDF 103 kb)

## Abbreviations

ART: Antiretroviral treatment
BP: Blood pressure
CBA: Controlled before-and-after
CCT: Controlled clinical trials
CDs: Communicable diseases
CEBHA+: Collaboration for evidence Based Healthcare and Public Health in Africa
CENTRAL: The Cochrane Central Register of Control Trials
CINHAL: Cumulative Index to Nursing and Allied Health Literature
EMBASE: Excerpta Medica Database
EPOC: Effective Practice and Organisation of Care
GRADE: Grading of Recommendations Assessment, Development and Evaluation
HbA1c: Glycated haemoglobin
HIV: Human Immuno-deficiency Virus
ICC: Intra-class correlation coefficient
ITS: Interrupted time series
LILACS: Literatura Latino-Americana e do Caribe em Ciênciasda Saúde
LMICs: Low- and middle income countries
MD: Mean difference
MEDLINE: Medical Literature Analysis and Retrieval System Online
NCDs: Non-communicable diseases
PHC: Primary healthcare
PLWH: People living with HIV
PRISMA: The Preferred Reporting Items for Systematic Reviews and Meta-Analyses
RCTs: Randomised controlled trials
RR: Risk ratio
SMD: Standardised mean difference
TB: Tuberculosis
TIDieR: Template for Interventions Description and Replication
WHO: World Health Organisation
