# Correction to: Integrated models of care for diabetes and hypertension in low- and middle-income countries (LMICs): Protocol for a systematic review

**DOI:** 10.1186/s13643-019-0943-6

**Published:** 2019-01-31

**Authors:** Jeannine Uwimana Nicol, Anke Rohwer, Taryn Young, Charlotte M Bavuma, Joerg J Meerpohl

**Affiliations:** 1 Centre for Evidence-Based Health Care, Division of Epidemiology and Biostatistics, Department of Global Health, Faculty of Medicine and Health Sciences, Stellenbosch University, Francie van Zijl drive, Parow, Cape Town, 7500 South Africa; 2 School of Public Health, College of Medicine and Health Science, University of Rwanda, Kicukiro, Kigali, Rwanda; 3 College of Medicine and Health Science, University of Rwanda, Kicukiro, Kigali, Rwanda; 4 Institute for Evidence in Medicine (for Cochrane Germany Foundation), Medical Center–University of Freiburg, Breisacher Strasse 153, 79110 Freiburg, Germany

**Correction to: Syst Rev (2018) 7:203**

**https://doi.org/10.1186/s13643-018-0865-8**

Following publication of the original article [1], the author reported that their family name was misspelled. The details are as follows:

**Incorrect name in the original article:**

Joerg J Meerphol

**Correct name:**

Joerg J Meerpohl

The original article has been corrected.
