# Supplementary material for: Integrated models of care for diabetes and hypertension in low- and middle-income countries (LMICs) : Protocol for a systematic review
Source: Syst Rev. 2018 Nov 20;7:203. doi: 10.1186/s13643-018-0865-8 (PMC6247752; doi:10.1186/s13643-018-0865-8)
Supplement: Supplementary file 2 — Search strategy for MEDLINE (PubMed). (PDF 103 kb) [file 13643_2018_865_MOESM2_ESM.pdf]

Additional file 2: Medline (PubMed) search strategy

#1 "Hypertension"[Mesh] OR (hypertension OR hypertention OR "blood pressure" OR “arterial pressure” OR systolic OR diastolic)[title/abstract]

#2 diabetes OR "diabetes mellitus")[title/abstract] OR "Diabetes Mellitus"[Mesh])

#3 #1 OR #2

#4 (dyslipidaemia OR dyslipidemia OR cholesterol OR LDL OR HDL OR triglyceride OR triglycerides OR low density lipoprotein OR high density lipoprotein OR low-density lipoprotein OR high-density lipoprotein)[title/abstract] OR "Dyslipidemias"[Mesh]

#5 ((((HIV OR hiv-1 OR hiv-2* OR hiv1 OR hiv2 OR hiv infect* OR human immunodeficiency virus OR human immune deficiency virus OR human immuno-deficiency virus OR human immune-deficiency virus OR ((human immun*) AND (deficiency virus)) OR acquired immunodeficiency syndromes OR acquired immune deficiency syndrome OR acquired immuno-deficiency syndrome OR acquired immune-deficiency syndrome OR ((acquired immun*) AND (deficiency syndrome)) OR HIV/AIDS)))) OR ((HIV infections [MeSH] OR HIV [MeSH]))

#6 (tuberculosis OR tuberculoses OR tb)[Title/Abstract] OR "tuberculosis"[Mesh]

#7 "noncommunicable disease" OR "noncommunicable diseases" OR "non-communicable disease" OR "non-communicable diseases" OR NCD OR NCDs OR "Noncommunicable Diseases"[Mesh]

#8 (comorbid* OR co-morbid* OR "co morbidity" OR multimorbidity OR multi-morbid OR "multi morbidity")[title/abstract] OR "Multimorbidity"[Mesh] OR "Comorbidity"[Mesh]

#9 multi-disease* OR multidisease* OR multi disease* OR multiple condition* OR multi-condition* OR multi condition* OR multiple illness* OR multi-illness* OR multi illness* OR multiple syndrome* OR multi-syndrome* OR multi syndrome* OR concurrent condition* OR concurrent illness* OR concurrent disease* OR co-existing disease* OR coexisting disease* OR co-existing illness* OR coexisting illness* OR co-existing syndrome* OR coexisting syndrome* OR co-existing condition* OR coexisting condition* OR co-occurring disease* OR co occuring disease* OR cooccuring disease* OR co-occurring illness* OR co occurring illness* OR cooccurring illness* OR co-occurring syndrome* OR co occurring syndrome* OR cooccurring syndrome* OR co-occurring condition* OR co occurring condition* OR cooccurring condition*

#10 chronic disease* OR lifestyle disease* OR "diseases of lifestyle" OR "disease of lifestyle" OR "Multiple Chronic Conditions"[Mesh] OR "Chronic Disease"[Mesh]

#11 #4 OR #5 OR #6 OR #7 OR #8 OR #9 OR #10

#12 "Delivery of Health Care, Integrated"[Mesh] OR “delivery of care” OR “delivery of health” OR "delivery of healthcare" OR "Comprehensive Health Care"[Mesh] OR "comprehensive healthcare" OR "comprehensive care" OR "comprehensive health" OR "Continuity of Patient Care"[Mesh] OR "continuity of patient care" OR "continuity of care" OR "continuity of health" OR "continuity of healthcare" OR "Patient-Centered Care"[Mesh] OR "patient centered care" OR "patient centred care"

#13 "Referral and Consultation"[Mesh] OR (referral AND consultation)

#14 integrat* care OR "integration of care" OR integrat* services OR "integration of services" OR integrat* programmes OR integrat* programs OR “integration of programmes” OR “integration of programs” OR integrat* service delivery OR “integration of service delivery” OR integrat* services OR “integration of services” OR integrat* delivery OR integrat* management OR “integration of management”

#15 coordinat* care OR "coordination of care" OR coordinat* services OR "coordination of services" OR coordinat* programmes OR coordinat* programs OR “coordination of programmes” OR “coordination of programs” OR coordinat* service delivery OR “coordination of service delivery” OR coordinat* services OR “coordination of services” OR coordinat* delivery OR coordinat* management OR “coordination of management”

#16 co-ordinat* care OR "co-ordination of care" OR co-ordinat* services OR "co-ordination of services" OR co-ordinat* programmes OR co-ordinat* programs OR “co-ordination of programmes” OR “co-ordination of programs” OR co-ordinat* service delivery OR “co-ordination of service delivery” OR co-ordinat* services OR “co-ordination of services” OR co-ordinat* delivery OR co-ordinat* management OR “co-ordination of management”

#17 horizontal care OR vertical care OR horizontal services OR vertical services OR horizontal programmes OR horizontal programs OR vertical programmes OR vertical programs OR horizontal service delivery OR vertical service delivery OR horizontal services OR vertical services OR horizontal delivery OR vertical management OR vertical management

#18 “multi team” OR multiteam “multi care” OR multicare OR “multi clinic” OR multiclinic OR “multi service” OR multiservice OR “multi program” OR multiprogram OR “multi programme” OR “multi delivery” OR multidelivery OR “multi management”

#19 #12 OR #13 OR #14 OR #15 OR #16 OR #17 OR #18

#20 #3 AND #11 AND #19

#21 Developing Countries[Mesh:noexp] OR Africa[Mesh:noexp] OR Africa, Northern[Mesh:noexp] OR Africa South of the Sahara[Mesh:noexp] OR Africa, Central[Mesh:noexp] OR Africa, Eastern[Mesh:noexp] OR Africa, Southern[Mesh:noexp] OR Africa, Western[Mesh:noexp] OR Asia[Mesh:noexp] OR Asia, Central[Mesh:noexp] OR Asia, Southeastern[Mesh:noexp] OR Asia, Western[Mesh:noexp] OR Caribbean Region[Mesh:noexp] OR West Indies[Mesh:noexp] OR South America[Mesh:noexp] OR Latin America[Mesh:noexp] OR Central America[Mesh:noexp] OR Afghanistan[Mesh:noexp] OR Albania[Mesh:noexp] OR Algeria[Mesh:noexp] OR American Samoa[Mesh:noexp] OR Angola[Mesh:noexp] OR "Antigua and Barbuda"[Mesh:noexp] OR Argentina[Mesh:noexp] OR Armenia[Mesh:noexp] OR Azerbaijan[Mesh:noexp] OR Bahrain[Mesh:noexp] OR Bangladesh[Mesh:noexp] OR Barbados[Mesh:noexp] OR Benin[Mesh:noexp] OR Byelarus[Mesh:noexp] OR Belize[Mesh:noexp] OR Bhutan[Mesh:noexp] OR Bolivia[Mesh:noexp] OR Bosnia-Herzegovina[Mesh:noexp] OR Botswana[Mesh:noexp] OR Brazil[Mesh:noexp] OR Bulgaria[Mesh:noexp] OR Burkina Faso[Mesh:noexp] OR Burundi[Mesh:noexp] OR Cambodia[Mesh:noexp] OR Cameroon[Mesh:noexp] OR Cape Verde[Mesh:noexp] OR Central African Republic[Mesh:noexp] OR Chad[Mesh:noexp] OR Chile[Mesh:noexp] OR China[Mesh:noexp] OR Colombia[Mesh:noexp] OR Comoros[Mesh:noexp] OR Congo[Mesh:noexp] OR Costa Rica[Mesh:noexp] OR Cote d'Ivoire[Mesh:noexp] OR Croatia[Mesh:noexp] OR Cuba[Mesh:noexp] OR Cyprus[Mesh:noexp] OR Czechoslovakia[Mesh:noexp] OR Czech Republic[Mesh:noexp] OR Slovakia[Mesh:noexp] OR Djibouti[Mesh:noexp] OR "Democratic Republic of the Congo"[Mesh:noexp] OR Dominica[Mesh:noexp] OR Dominican Republic[Mesh:noexp] OR East Timor[Mesh:noexp] OR Ecuador[Mesh:noexp] OR Egypt[Mesh:noexp] OR El Salvador[Mesh:noexp] OR Eritrea[Mesh:noexp] OR Estonia[Mesh:noexp] OR Ethiopia[Mesh:noexp] OR Fiji[Mesh:noexp] OR Gabon[Mesh:noexp] OR Gambia[Mesh:noexp] OR "Georgia (Republic)"[Mesh:noexp] OR Ghana[Mesh:noexp] OR Greece[Mesh:noexp] OR Grenada[Mesh:noexp] OR Guatemala[Mesh:noexp] OR Guinea[Mesh:noexp] OR Guinea-Bissau[Mesh:noexp] OR Guam[Mesh:noexp] OR Guyana[Mesh:noexp] OR Haiti[Mesh:noexp] OR Honduras[Mesh:noexp] OR Hungary[Mesh:noexp] OR India[Mesh:noexp] OR Indonesia[Mesh:noexp] OR Iran[Mesh:noexp] OR Iraq[Mesh:noexp] OR

Jamaica[Mesh:noexp] OR Jordan[Mesh:noexp] OR Kazakhstan[Mesh:noexp] OR Kenya[Mesh:noexp] OR Korea[Mesh:noexp] OR Kosovo[Mesh:noexp] OR Kyrgyzstan[Mesh:noexp] OR Laos[Mesh:noexp] OR Latvia[Mesh:noexp] OR Lebanon[Mesh:noexp] OR Lesotho[Mesh:noexp] OR Liberia[Mesh:noexp] OR Libya[Mesh:noexp] OR Lithuania[Mesh:noexp] OR Macedonia[Mesh:noexp] OR Madagascar[Mesh:noexp] OR Malaysia[Mesh:noexp] OR Malawi[Mesh:noexp] OR Mali[Mesh:noexp] OR Malta[Mesh:noexp] OR Mauritania[Mesh:noexp] OR Mauritius[Mesh:noexp] OR Mexico[Mesh:noexp] OR Micronesia[Mesh:noexp] OR Middle East[Mesh:noexp] OR Moldova[Mesh:noexp] OR Mongolia[Mesh:noexp] OR Montenegro[Mesh:noexp] OR Morocco[Mesh:noexp] OR Mozambique[Mesh:noexp] OR Myanmar[Mesh:noexp] OR Namibia[Mesh:noexp] OR Nepal[Mesh:noexp] OR Netherlands Antilles[Mesh:noexp] OR New Caledonia[Mesh:noexp] OR Nicaragua[Mesh:noexp] OR Niger[Mesh:noexp] OR Nigeria[Mesh:noexp] OR Oman[Mesh:noexp] OR Pakistan[Mesh:noexp] OR Palau[Mesh:noexp] OR Panama[Mesh:noexp] OR Papua New Guinea[Mesh:noexp] OR Paraguay[Mesh:noexp] OR Peru[Mesh:noexp] OR Philippines[Mesh:noexp] OR Poland[Mesh:noexp] OR Portugal[Mesh:noexp] OR Puerto Rico[Mesh:noexp] OR Romania[Mesh:noexp] OR Russia[Mesh:noexp] OR "Russia (Pre-1917)"[Mesh:noexp] OR Rwanda[Mesh:noexp] OR "Saint Kitts and Nevis"[Mesh:noexp] OR Saint Lucia[Mesh:noexp] OR "Saint Vincent and the Grenadines"[Mesh:noexp] OR Samoa[Mesh:noexp] OR Saudi Arabia[Mesh:noexp] OR Senegal[Mesh:noexp] OR Serbia[Mesh:noexp] OR Montenegro[Mesh:noexp] OR Seychelles[Mesh:noexp] OR Sierra Leone[Mesh:noexp] OR Slovenia[Mesh:noexp] OR Sri Lanka[Mesh:noexp] OR Somalia[Mesh:noexp] OR South Africa[Mesh:noexp] OR Sudan[Mesh:noexp] OR Suriname[Mesh:noexp] OR Swaziland[Mesh:noexp] OR Syria[Mesh:noexp] OR Tajikistan[Mesh:noexp] OR Tanzania[Mesh:noexp] OR Thailand[Mesh:noexp] OR Togo[Mesh:noexp] OR Tonga[Mesh:noexp] OR "Trinidad and Tobago"[Mesh:noexp] OR Tunisia[Mesh:noexp] OR Turkey[Mesh:noexp] OR Turkmenistan[Mesh:noexp] OR Uganda[Mesh:noexp] OR Ukraine[Mesh:noexp] OR Uruguay[Mesh:noexp] OR USSR[Mesh:noexp] OR Uzbekistan[Mesh:noexp] OR Vanuatu[Mesh:noexp] OR Venezuela[Mesh:noexp] OR Vietnam[Mesh:noexp] OR Yemen[Mesh:noexp] OR Yugoslavia[Mesh:noexp] OR Zambia[Mesh:noexp] OR Zimbabwe[Mesh:noexp]

#22 Macedonia[tw] OR Madagascar[tw] OR Malagasy Republic[tw] OR Malaysia[tw] OR Malaya[tw] OR Malay[tw] OR Sabah[tw] OR Sarawak[tw] OR Malawi[tw] OR Nyasaland[tw] OR Mali[tw] OR Malta[tw] OR Marshall Islands[tw] OR Mauritania[tw] OR Mauritius[tw] OR Agalega Islands[tw] OR Mexico[tw] OR Micronesia[tw] OR Middle East[tw] OR Moldova[tw] OR Moldovia[tw] OR Moldovian[tw] OR Mongolia[tw] OR Montenegro[tw] OR Morocco[tw] OR Ifni[tw] OR Mozambique[tw] OR Myanmar[tw] OR Myanma[tw] OR Burma[tw] OR Namibia[tw] OR Nepal[tw] OR Netherlands Antilles[tw] OR New Caledonia[tw] OR Nicaragua[tw] OR Niger[tw] OR Nigeria[tw] OR Northern Mariana Islands[tw] OR Oman[tw] OR Muscat[tw] OR Pakistan[tw] OR Palau[tw] OR Palestine[tw] OR Panama[tw] OR Paraguay[tw] OR Peru[tw] OR Philippines[tw] OR Philipines[tw] OR Phillipines[tw] OR Phillippines[tw] OR Poland[tw] OR Portugal[tw] OR Puerto Rico[tw] OR Romania[tw] OR Rumania[tw] OR Roumania[tw] OR Russia[tw] OR Russian[tw] OR Rwanda[tw] OR Ruanda[tw] OR Saint Kitts[tw] OR St Kitts[tw] OR Nevis[tw] OR Saint Lucia[tw] OR St Lucia[tw] OR Saint Vincent[tw] OR St Vincent[tw] OR Grenadines[tw] OR Samoa[tw] OR Samoan Islands[tw] OR Navigator Island[tw] OR Navigator Islands[tw] OR Sao Tome[tw] OR Saudi Arabia[tw] OR Senegal[tw] OR Serbia[tw] OR Montenegro[tw] OR Seychelles[tw] OR Sierra Leone[tw] OR Slovenia[tw] OR Sri Lanka[tw] OR Ceylon[tw] OR Solomon Islands[tw] OR Somalia[tw] OR Sudan[tw] OR Suriname[tw] OR Surinam[tw] OR Swaziland[tw] OR Syria[tw] OR Tajikistan[tw] OR Tadzhikistan[tw] OR Tadjikistan[tw] OR Tadzhik[tw] OR Tanzania[tw] OR Thailand[tw] OR Togo[tw] OR Togolese Republic[tw] OR Tonga[tw] OR Trinidad[tw] OR Tobago[tw] OR Tunisia[tw] OR Turkey[tw] OR Turkmenistan[tw] OR Turkmen[tw] OR Uganda[tw] OR Ukraine[tw] OR Uruguay[tw] OR USSR[tw] OR Soviet Union[tw] OR Union of Soviet Socialist Republics[tw] OR Uzbekistan[tw] OR Uzbek OR Vanuatu[tw] OR New

Hebrides[tw] OR Venezuela[tw] OR Vietnam[tw] OR Viet Nam[tw] OR West Bank[tw] OR Yemen[tw] OR Yugoslavia[tw] OR Zambia[tw] OR Zimbabwe[tw] OR Rhodesia[tw]

#23 Africa[tw] OR Asia[tw] OR Caribbean[tw] OR West Indies[tw] OR South America[tw] OR Latin America[tw] OR Central America[tw] OR Afghanistan[tw] OR Albania[tw] OR Algeria[tw] OR Angola[tw] OR Antigua[tw] OR Barbuda[tw] OR Argentina[tw] OR Armenia[tw] OR Armenian[tw] OR Aruba[tw] OR Azerbaijan[tw] OR Bahrain[tw] OR Bangladesh[tw] OR Barbados[tw] OR Benin[tw] OR Byelarus[tw] OR Byelorussian[tw] OR Belarus[tw] OR Belorussian[tw] OR Belorussia[tw] OR Belize[tw] OR Bhutan[tw] OR Bolivia[tw] OR Bosnia[tw] OR Herzegovina[tw] OR Hercegovina[tw] OR Botswana[tw] OR Brasil[tw] OR Brazil[tw] OR Bulgaria[tw] OR Burkina Faso[tw] OR Burkina Fasso[tw] OR Upper Volta[tw] OR Burundi[tw] OR Urundi[tw] OR Cambodia[tw] OR Khmer Republic[tw] OR Kampuchea[tw] OR Cameroon[tw] OR Cameroons[tw] OR Cameron[tw] OR Camerons[tw] OR Cape Verde[tw] OR Central African Republic[tw] OR Chad[tw] OR Chile[tw] OR China[tw] OR Colombia[tw] OR Comoros[tw] OR Comoro Islands[tw] OR Comores[tw] OR Mayotte[tw] OR Congo[tw] OR Zaire[tw] OR Costa Rica[tw] OR Cote d'Ivoire[tw] OR Ivory Coast[tw] OR Croatia[tw] OR Cuba[tw] OR Cyprus[tw] OR Czechoslovakia[tw] OR Czech Republic[tw] OR Slovakia[tw] OR Slovak Republic[tw] OR Djibouti[tw] OR French Somaliland[tw] OR Dominica[tw] OR Dominican Republic[tw] OR East Timor[tw] OR East Timur[tw] OR Timor Leste[tw] OR Ecuador[tw] OR Egypt[tw] OR United Arab Republic[tw] OR El Salvador[tw] OR Eritrea[tw] OR Estonia[tw] OR Ethiopia[tw] OR Fiji[tw] OR Gabon[tw] OR Gabonese Republic[tw] OR Gambia[tw] OR Gaza[tw] OR Georgia Republic[tw] OR Georgian Republic[tw] OR Ghana[tw] OR Gold Coast[tw] OR Greece[tw] OR Grenada[tw] OR Guatemala[tw] OR Guinea[tw] OR Guam[tw] OR Guiana[tw] OR Guyana[tw] OR Haiti[tw] OR Honduras[tw] OR Hungary[tw] OR India[tw] OR Maldives[tw] OR Indonesia[tw] OR Iran[tw] OR Iraq[tw] OR Isle of Man[tw] OR Jamaica[tw] OR Jordan[tw] OR Kazakhstan[tw] OR Kazakh[tw] OR Kenya[tw] OR Kiribati[tw] OR Korea[tw] OR Kosovo[tw] OR Kyrgyzstan[tw] OR Kirghizia[tw] OR Kyrgyz Republic[tw] OR Kirghiz[tw] OR Kirgizstan[tw] OR "Lao PDR"[tw] OR Laos[tw] OR Latvia[tw] OR Lebanon[tw] OR Lesotho[tw] OR Basutoland[tw] OR Liberia[tw] OR Libya[tw] OR Lithuania[tw]

#24 "developing country"[tw] OR "developing countries"[tw] OR "developing nation"[tw] OR "developing nations"[tw] OR "developing population"[tw] OR "developing populations"[tw] OR "developing world"[tw] OR "less developed country"[tw] OR "less developed countries"[tw] OR "less developed nation"[tw] OR "less developed nations"[tw] OR "less developed population"[tw] OR "less developed populations"[tw] OR "less developed world"[tw] OR "lesser developed country"[tw] OR "lesser developed countries"[tw] OR "lesser developed nation"[tw] OR "lesser developed nations"[tw] OR "lesser developed population"[tw] OR "lesser developed populations"[tw] OR "lesser developed world"[tw] OR "under developed country"[tw] OR "under developed countries"[tw] OR "under developed nation"[tw] OR "under developed nations"[tw] OR "under developed population"[tw] OR "under developed populations"[tw] OR "under developed world"[tw] OR "underdeveloped country"[tw] OR "underdeveloped countries"[tw] OR "underdeveloped nation"[tw] OR "underdeveloped nations"[tw] OR "underdeveloped population"[tw] OR "underdeveloped populations"[tw] OR "underdeveloped world"[tw] OR "middle income country"[tw] OR "middle income countries"[tw] OR "middle income nation"[tw] OR "middle income nations"[tw] OR "middle income population"[tw] OR "middle income populations"[tw] OR "low income country"[tw] OR "low income countries"[tw] OR "low income nation"[tw] OR "low income nations"[tw] OR "low income population"[tw] OR "low income populations"[tw] OR "lower income country"[tw] OR "lower income countries"[tw] OR "lower income nation"[tw] OR "lower income nations"[tw] OR "lower income population"[tw] OR "lower income populations"[tw] OR "underserved country"[tw] OR "underserved countries"[tw] OR "underserved nation"[tw] OR "underserved nations"[tw] OR "underserved population"[tw] OR "underserved populations"[tw] OR "underserved world"[tw] OR "under served country"[tw] OR "under served countries"[tw] OR "under served nation"[tw] OR "under served nations"[tw] OR "under served population"[tw] OR "under served populations"[tw] OR "under served

world"[tw] OR "deprived country"[tw] OR "deprived countries"[tw] OR "deprived nation"[tw] OR "deprived nations"[tw] OR "deprived population"[tw] OR "deprived populations"[tw] OR "deprived world"[tw] OR "poor country"[tw] OR "poor countries"[tw] OR "poor nation"[tw] OR "poor nations"[tw] OR "poor population"[tw] OR "poor populations"[tw] OR "poor world"[tw] OR "poorer country"[tw] OR "poorer countries"[tw] OR "poorer nation"[tw] OR "poorer nations"[tw] OR "poorer population"[tw] OR "poorer populations"[tw] OR "poorer world"[tw] OR "developing economy"[tw] OR "developing economies"[tw] OR "less developed economy"[tw] OR "less developed economies"[tw] OR "lesser developed economy"[tw] OR "lesser developed economies"[tw] OR "under developed economy"[tw] OR "under developed economies"[tw] OR "underdeveloped economy"[tw] OR "underdeveloped economies"[tw] OR "middle income economy"[tw] OR "middle income economies"[tw] OR "low income economy"[tw] OR "low income economies"[tw] OR "lower income economy"[tw] OR "lower income economies"[tw] OR "low gdp"[tw] OR "low gnp"[tw] OR "low gross domestic"[tw] OR "low gross national"[tw] OR "lower gdp"[tw] OR "lower gnp"[tw] OR "lower gross domestic"[tw] OR "lower gross national"[tw] OR lmic[tw] OR lmics[tw] OR "third world"[tw] OR "lami country"[tw] OR "lami countries"[tw] OR "transitional country"[tw] OR "transitional countries"[tw]

#25 #21 OR #22 OR #23 OR #24

#26 #20 AND #25
